# Interbrain connectivity during mindfulness and meditation: narrative review of hyperscanning research

**DOI:** 10.3389/fcogn.2026.1719931

**Published:** 2026-04-13

**Authors:** Lorne Schussel

**Affiliations:** Department of Counseling and Clinical Psychology, Teachers College, Columbia University, New York, NY, United States

**Keywords:** hyperscanning, interbrain synchrony, meditation, mindfulness, social neuroscience

## Abstract

Over the past century, research on meditation and mindfulness has aimed to characterize the behavioral phenomenology and the neurophysiology of the contemplative mind. In recent years, investigators have extended this work beyond single participants to dyads and larger groups using hyperscanning (the simultaneous recording of neural activity from two or more individuals). This narrative review synthesizes interbrain connectivity research and explores dyadic synchrony findings within mindfulness and meditation practices. Among the selected studies (*n* = 7), interbrain synchronization was observed across protocols and spectral bands in five studies. Anterior theta phase synchrony was evident during cooperation tasks following mindfulness induction. Motor coordination tasks with shared breathing and physical mirroring elicited alpha, theta, and delta coherence in frontal brain regions. Gamma synchrony increased in socio-emotional paradigms and among expert meditators practicing together. Dyadic coupling in lower-frequency spectral bands was potentiated when breath focus was combined with a shared goal. The evidence reviewed suggests that interbrain synchrony varies as a function of expertise, task heterogeneity, and personality traits such as agreeableness.

## Introduction

In Western philosophical rationalism, Spinoza proposed that all beings are unified as one substance and that individuals are not isolated but rather are manifestations of an underlying reality. This statement is echoed in Eastern philosophy and contemporary mindfulness discourse. One of the founders of modern mindfulness, Thich Nhat Hanh, writes, “*We are here to awaken from the illusion of our separateness*.” The contemporary quote reflects Spinoza's philosophical monism, merging Eastern and Western perspectives and alluding to a connective substrate that unifies all beings (Spinoza, 1677/1996; Thich Nhat Hanh, 1998a,b).

This notion of an inherent interconnectedness has increasingly informed empirical work in contemplative science and mindfulness research. Over the past two decades, a body of research has demonstrated robust effects across biophysiological and psychological domains, including reductions in anxiety and depressive symptoms, improvements in overall wellbeing, enhanced attention, and strengthened executive function (Carlson et al., 2007; Fredrickson et al., 2008; Goyal et al., 2014; Jha et al., 2007; Segal et al., 2010; Tang et al., 2007).

Neurophysiological findings posit self-induced modulation of attention and executive control systems, changes in spectral power across frequency bands, alterations in event-related potential latency, changes in neural coherence, and interbrain synchrony (Brewer et al., 2011; Tang et al., 2015; Goyal et al., 2014; Cahn and Polich, 2006; Fox et al., 2016; Lutz et al., 2004). Other changes include intrinsic brain connectivity, social connectivity, and enhanced closeness between individuals (Carson et al., 2004; Hutcherson et al., 2008; Kok and Singer, 2017; Lindsay et al., 2019).

These studies provide a scientific scaffold for examining how contemplative practices manifest biophysiological changes. Some allude to the “connection” that both Eastern and Western philosophical traditions describe and that has intrigued humanity for centuries. Hyperscanning is now able to broach this nascent research domain.

### Hyperscanning empirical findings

Hyperscanning refers to the simultaneous recording of neural activity from two or more individuals and in some cases includes nonneural physiological inputs, with data typically acquired through magnetic resonance imaging (MRI), electroencephalography (EEG), or functional near-infrared spectroscopy (fNIRS). The approach allows researchers to investigate how individuals coordinate attention, exchange information during shared activities, and participate in joint action within behavioral paradigms. Connectivity outcomes are generally indexed through measures of interbrain synchrony, often described as time-locked and frequency band–specific coupling (Dumas et al., 2010; Hasson et al., 2012; Montague et al., 2002; Koike et al., 2016). Over the past four decades, this method has been applied across a wide range of experimental paradigms and naturalistic settings (Montague et al., 2002).

A significant portion of hyperscanning studies is linked to game theory paradigms, decision-making processes, cooperative joint action, and negative feedback tasks. Far fewer studies are based on music, simulated training, behavioral mimicry, and differential tasks in naturalistic settings such as attending a concert or museum (Astolfi et al., 2011; Babiloni et al., 2012; Dikker et al., 2017; Dumas et al., 2010; Lindenberger et al., 2009; Sänger et al., 2012).

During most game theory study paradigms, researchers use primarily the prisoner's dilemma, the ultimatum game, and the dictator game (Astolfi et al., 2010; Balconi and Vanutelli, 2017; Cui et al., 2012; King-Casas et al., 2005; Montague et al., 2002; TenHouten et al., 2023; Noah et al., 2020). Cooperative decision-making tasks exhibited greater synchrony in the frontal gyrus, prefrontal cortex (PFC), and temporal parietal junction, and these brain regions are associated with social cognition and perspective formation. Within the EEG component of the studies, alpha and theta bands were the most identified spectral windows for synchrony (Astolfi et al., 2010; Balconi and Vanutelli, 2017; Dikker et al., 2017; Lindenberger et al., 2009; Montague et al., 2002).

In the last 8 years, several studies have been based on classroom learning, counseling, and mindfulness modalities. Recent work with larger arrays of lower-density EEGs has been implemented in the classroom with shared listening and participation in an educational setting with unique research outcomes (Dikker et al., 2017; Bevilacqua et al., 2019). In a classroom study of interbrain synchrony, where a cohort of students collectively listen to the same lesson, synchrony was observed with EEG in alpha and beta bands and temporally matched the teacher's classroom instruction (Dikker et al., 2017). Higher synchrony related to moments of collective engagement (coded student observation and student self-report) was predictive of better memory and learning outcomes.

In a comparable study using interparticipant MRI coupling, researchers measured a shared learning and listening task through a storytelling process. Study participants listened to a story and formed an internal shared narrative. During periods of measured comprehension there was intrabrain synchrony between storyteller and study participants found within the temporal cortex and frontal brain regions (Liu et al., 2018). Shared internal cognitive models through language have resurfaced in recent studies within counseling and therapy-based analogs of hyperscanning. Zhang et al. (2020) examined synchrony *in vivo* during talk therapy sessions between different student–counselor dyads. The study found neural synchrony in the temporal parietal junction between the counselor and student that was correlated with higher therapeutic alliance scores. The relationship was absent in the nontherapeutic “chatting” control group (Zhang et al., 2020).

In a hypercanning study of counseling that examined both shared meaning and communication, Dai et al. (2023) posited that the timing of synchrony during the session was important and predictive of therapeutic outcomes in the session. For example, early session interbrain synchrony localized in the right angular gyrus and temporal parietal junction predicted attenuation of psychological distress. The authors suggest that shared perspective and social alignment temporally can have predictive value for pathology and provide mechanistic evidence for how shared states manifest in different group dynamics.

If shared attention and shared semantics can connect individual brains, perhaps mindfulness, a practice of unified presence, may also rely on overlapping mechanisms of interpersonal and social alignment. This paper aims to elucidate this possible connection. Newly formed empirical support of this concept comes from studies showing synchrony in mindfulness and contemplative modalities. In a study of adult dyads using a mindfulness and cooperation paradigm, interbrain synchrony increased in frontal theta bands when compared to a rest period. When there was a shared experience of mutual success with respective success feedback, synchrony was higher between adult dyads including a higher phase-locking value (PLV) in the theta band (Deng et al., 2023). In a mindfulness and breath-focused task with interoceptive breath awareness and coordinated motor skills (dual hand movements), combined with a shared goal task, there was a significant increase in interbrain synchrony in delta, theta, and alpha bands (Balconi and Angioletti, 2024). While these findings represent a small sample and recent extension of hyperscanning into contemplative science, they are part of a larger framework of interbrain research within the cognitive science literature and will be further synthesized.

## Methods

A narrative review was implemented to examine hyperscanning research investigating human connectivity within mindfulness and meditation practices. Although this manuscript follows a narrative mini-review format, study identification and screening were informed by Preferred Reporting Items for Systematic Reviews and Meta-Analyses (PRISMA) guidelines to increase transparency and for additional methodological rigor. Searches were implemented through PubMed, MEDLINE, ScienceDirect, and PubMed Central. Additionally, targeted handsearches of major publisher platforms (e.g., Nature Portfolio, Scientific Reports, Frontiers) were conducted to ensure that no relevant peer-reviewed records were missed, due to the small and nascent body of hyperscanning literature. Only peer-reviewed and English language papers were considered. Reference lists of the included studies were also manually screened to identify any additional eligible articles. The search used the following Boolean strings that combined hyperscanning terms: “hyperscanning,” “interbrain,” “brain-to-brain” with contemplative terms “mindfulness,” “meditation,” “open monitoring,” “focused attention,” “interoception,” “breath.” After deduplication, titles and abstracts were screened for human studies that used simultaneous dual EEG, fNIRS, or EEG–fNIRS in dyads or multisubject groups during a protocol induction (guided mindfulness), (task embedded interoceptive manipulation via breath focus). Screening and eligibility assessment were conducted by a single reviewer due to the narrative mini-review format and the limited size of the evidence base. The search was conducted in October 2025 and includes all records available to that date. Due to increasing methodological heterogeneity and the small subset of eligible papers identified, a meta-analysis was not undertaken.

**Figure 1:**
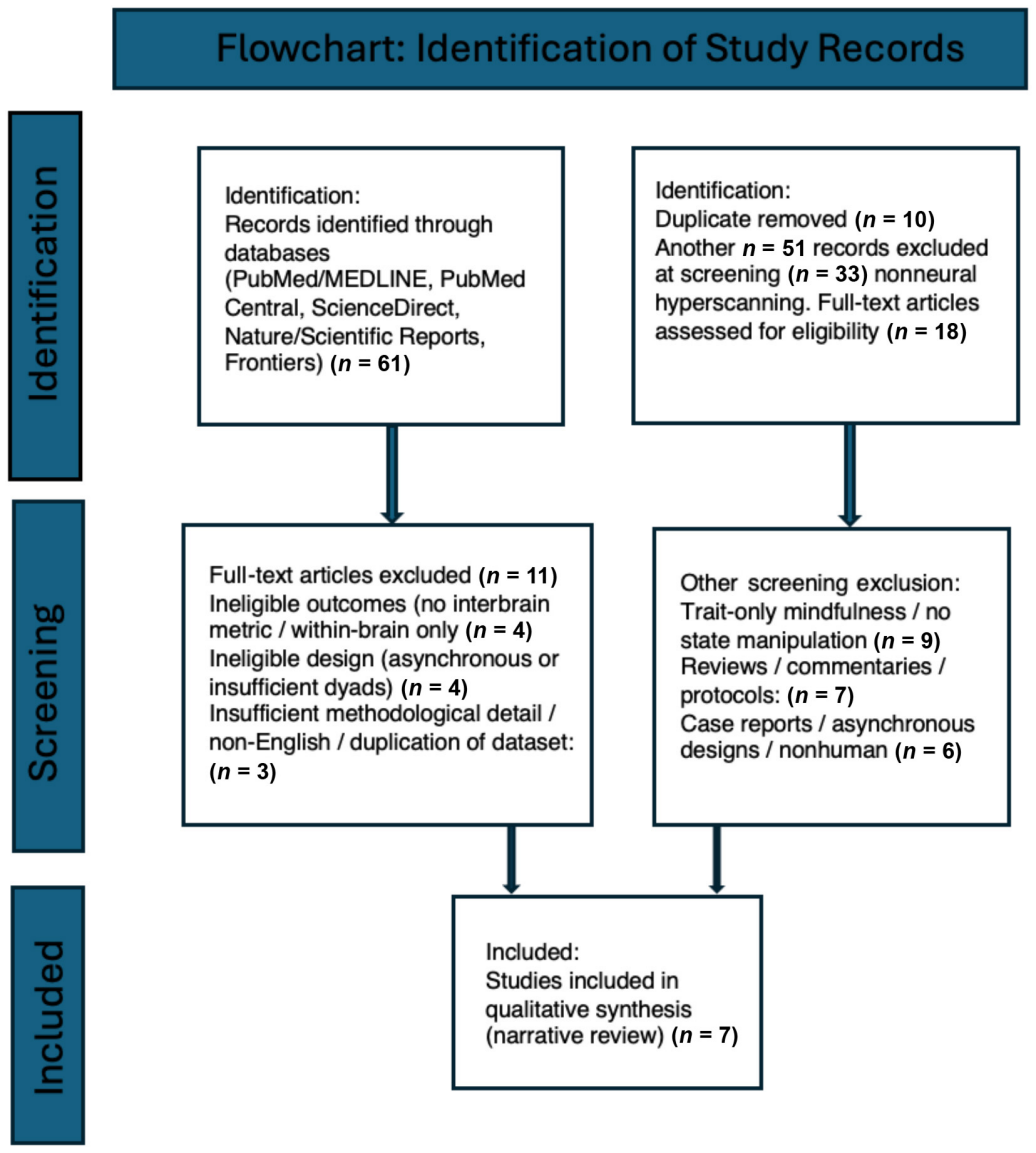
Study identification, screening, eligibility, and inclusion for hyperscanning mindfulness review. Records identified (*n* = 61), duplicates removed (*n* = 10), screened (*n* = 51), excluded with reasons (*n* = 33), full texts assessed (*n* = 18), excluded with reasons (*n* = 11), and studies included (*n* = 7). Adapted from PRISMA 2020 (Page et al., 2021).

Eligibility criteria included human participants, simultaneous neural data collection of two or more individuals, an active intervention, or task-based experimental manipulation. For active intervention or task manipulation, studies were included if they involved a guided mindfulness or meditation intervention or an interoceptive breath-focused manipulation. Studies were excluded if they examined trait mindfulness only without any specified intervention or were reviews or commentaries on meditation or other mindfulness work. Also, single case studies were excluded, along with papers that had nonneural comparisons and asynchronous recordings. Also removed were trait-only mindfulness studies without interventions, reviews, or commentaries, single case reports, nonneural synchrony, and asynchronous recordings. Although a narrative mini-review was used, a PRISMA flow diagram log is included to show search inquiry parameters that supported search, including final documents identified, screened eligibility, and inclusion papers (Page et al., 2021). Abstracts and titles were further screened before further review, and concerns regarding eligibility were addressed through re-examination of inclusion criteria (Figure 1).

Imported studies were systematically included across various bodies of literature and domains including participants characteristics via age, level of expertise, practice types, intervention duration, task context via cooperative feedback, motor-based or linguistic synchrony, shared affect, neural modality, acquisition region of interest, and synchrony metrics (Bastos and Schoffelen, 2016; Delorme and Makeig, 2004; Gramfort et al., 2014; Lachaux et al., 1999; Nolte et al., 2004).

In addition to the study identification pipeline, methodological quality and risk of bias were further assessed. Randomized designs were assessed using Cochrane Risk of Bias 2 (RoB 2), and nonrandomized designs were examined using the National Institutes of Health Quality Assessment Tool. With respect to the unique aspects of hyperscanning, domain-specific criteria considered synchronization fidelity, shared sensory input, and artifact management. Across the studies, risk of bias ranged from low to fair (Sterne et al., 2019; National Heart, Lung, and Blood Institute, 2014). There were small sample sizes and inconsistent application of the surrogate or time-shift null controls. Study strengths include clearly defined task contrasts, counterbalanced designs, and, in one specific study, a surrogate-dyad null model (Deng et al., 2023).

### Data organization

Given heterogeneity and differences between various experimental paradigms, the findings were addressed through analytic approaches by organizing frequency domains (delta, theta, alpha, and gamma), spatial locality, task and meditation type, comparison groups, and directionality of results via synchrony or connectivity metrics. Data was further categorized by spatial localization across frontal, central, temporal, parietal, occipital, and limbic regions (Balconi and Angioletti, 2023; Coomans et al., 2021; Deng et al., 2023, 2024; Matiz et al., 2021). Aggregate data were also organized based on the context of a task used for the experimental adjunct to mindfulness. Multiple tasks and interventions were included in the review. There were several unique meditations and breath-based modalities such as synchronous interoceptive breathing with behavioral synchrony via coordination of several motor tasks. For example, participants would sit side by side and use hand movements in direct mirroring of one another. During hand movements, participants mindfully focused on their breathing (Balconi and Angioletti, 2023, 2024). In a related crossover design, interoceptive breath focus with an emphasis on shared goals of succeeding together was employed as the study task. Another paradigm was examining joint meditation participation vs. individual practice (Coomans et al., 2021). Additional designs include a brief and guided mindfulness experience incorporating a body scan and breath awareness, followed by several cooperative feedback tasks in adult populations (Deng et al., 2023) and emotional picture viewing in adolescents (Deng et al., 2024). Finally, a classroom-based meditation with musical and resting-state comparison groups (Gao et al., 2025). Body scan–based meditation, breath-based mindfulness with emotionally laden content, and motor-based coordination with breath focus were synthesized into a collective framework and organized into the narrative review. What follows is a summary table for mindfulness modalities used across the seven studies that outlines specific protocols for clarity (Table 1).

**Table 1:** Meditation, mindfulness, and interoceptive practices within included studies.

Practice type	Description of practice	Included
Brief guided mindfulness (breath-centered + body scan) prior to a social task (cooperation or shared emotional viewing activity)	Short audio-guided mindfulness induction combining breath-focused attention and a body-scan practice, emphasizing nonjudgmental awareness of breathing and bodily sensations prior to a social interaction task (cooperation or shared emotional viewing activity).	Deng et al., 2023, 2024
Interoceptive breath focus (task-embedded)	Participants were instructed to direct attention to their breath while performing coordination tasks. Breath-focused attention was embedded within the task and not delivered as a stand-alone meditation.	Balconi and Angioletti, 2023
Interoceptive breath focus with shared goal (EEG–fNIRS)	Breath focus is used during dyadic motor synchrony with an added manipulation of shared goal/intentional coordination; multimodal EEG–fNIRS hyperscanning emphasizes aPFC entrainment.	Balconi and Angioletti, 2024
Joint vs. individual mindful breathing (dyadic co-presence manipulation)	Focused-attention breathing is performed either jointly (dyad co-present) or individually (screen separation), enabling a direct test of co-presence effects on coherence; trait moderation is tested.	Coomans et al., 2021
Open-monitoring meditation (experts) vs. instructed mind wandering; together vs. apart	Experienced meditators perform open monitoring vs. instructed mind wandering in same-room vs. different-room conditions; analyzed via IC cluster power (co-activation).	Matiz et al., 2021
Classroom breath-based meditation (group)	Short group seated breath meditation is compared with rest and music and assessed via single-channel alpha correlation and network topology metrics.	Gao et al., 2025

In addition to measuring shared or composite effects from the study, Table 2 below highlights connectivity metrics for the small sample of studies that were part of the literature. Synchrony metrics included in the paper were quantified using frequency and time domain comparisons with EEG coherence (degree to which signals oscillate and align in a specified frequency range and derived from the cross-spectral density between two signals). Also, the PLV was included and is a measure of relative wave differences or phase synchrony between the composite signal in two different sets of electrodes or brain regions. Other metrics of coherence, such as partial-correlation coherence, were also included as an alternate estimate that makes the coherence calculation by removing shared variance and controls for a common source to give an estimate with reduced error and account for inflated readings (Nolte et al., 2004; Maris and Oostenveld, 2007). In Balconi and Angioletti (2024), within-EEG and fNIRS data synchrony was assessed using cross-modal variations to compare electrophysiological and hemodynamic synchrony. Interbrain synchrony was computed with the different study pairs using correlation-centered indices for capturing entrainment within slower frequency windows known to be associated to metabolic activity. Table 2 further summarizes the mathematical models and synchrony methods used to evaluate hyperscanning and neural connectivity data, including those used in this review and others commonly applied in the wider neural connectivity subfield.

**Table 2:** Common interbrain connectivity metrics used in hyperscanning studies.

Pearson correlation: $r_{xy}=\frac{\overset{T}{\underset{t=1}{\sum }}\left(x_{t}-\bar{x}\right)\left(y_{t}-\bar{y}\right)}{\sqrt{\overset{T}{\underset{t=1}{\sum }}{\left(x_{t}-\bar{x}\right)}^{2}\overset{T}{\underset{t=1}{\sum }}{\left(y_{t}-\bar{y}\right)}^{2}}}$	Pearson correlation quantifies amplitude covariation between two neural time series. In hyperscanning, it is typically applied to band-limited power to estimate coupling between individuals (Cohen, 2014; Burgess, 2013).
Spectral coherence: $C_{xy}\left(f\right)=\frac{|S_{xy}\left(f\right){|}^{2}}{S_{xx}\left(f\right)S_{yy}\left(f\right)}$	Spectral coherence measures frequency-specific coupling based on cross-spectral density. It reflects joint amplitude phase alignment within an oscillatory band and is widely used in EEG hyperscanning (Bastos and Schoffelen, 2016; Nolte et al., 2004).
Phase-locking value (PLV): $PLV_{xy}=|\frac{1}{N}\overset{N}{\underset{k=1}{\sum }}e^{i\left(\phi_{x}\left(k\right)-\phi_{y}\left(k\right)\right)}|$	PLV assesses how stable the phase difference between two signals remains across time or across repeated trials (Lachaux et al., 1999).
Power envelope correlation: $r_{P_{x}P_{y}}where\;P_{x}\left(f\right)=|X\left(f\right){|}^{2}$	Power envelope correlation examines whether fluctuations in band-limited power covary between individuals (Bastos and Schoffelen, 2016).
Partial coherence: $C_{xy|z}\left(f\right)=\frac{{|S_{xy}\left(f\right)-\frac{S_{xz}\left(f\right)S_{zy}\left(f\right)}{S_{zz}\left(f\right)}|}^{2}}{\left(S_{xx}\left(f\right)-\frac{|S_{xz}\left(f\right){|}^{2}}{S_{zz}\left(f\right)}\right)\left(S_{yy}\left(f\right)-\frac{|S_{yz}\left(f\right){|}^{2}}{S_{zz}\left(f\right)}\right)}$	Partial coherence estimates frequency-specific coupling after removing variance shared with a third signal, a more conservative estimate (Nolte et al., 2004).
Imaginary coherence: ICxy(f)=Im(Sxy(f))Sxx(f)Syy(f)	Imaginary coherence isolates the imaginary component of the cross-spectrum, minimizing artifacts that may arise from shared sources. This approach is often employed to protect against spurious synchrony in EEG–based hyperscanning (Nolte et al., 2004).
Granger causality (time domain): $y_{t}=\overset{p}{\underset{k=1}{\sum }}a_{k}y_{t-k}+\overset{p}{\underset{k=1}{\sum }}b_{k}x_{t-k}+\varepsilon_{t}$	Time-domain Granger causality evaluates whether past activity in one signal improves prediction of another signal's future activity. Unlike undirected synchrony metrics, it estimates directional influence (Geweke, 1982; Granger, 1969; Seth et al., 2015).
Frequency-domain granger: $F_{x\to y}\left(f\right)=\ln\left(\frac{S_{yy}\left(f\right)}{S_{yy}\left(f\right)-|H_{yx}\left(f\right){|}^{2}S_{xx}\left(f\right)}\right)$	Frequency-domain Granger causality decomposes directional influence across oscillatory bands. It integrates spectral analysis with autoregressive modeling to estimate lagged interactions within specific frequency ranges (Geweke, 1982; Granger, 1969; Seth et al., 2015).

One additional metric mentioned in the table is Granger causality. In the case of meditators in dyadic conditions, such a methodology could determine whether a single individual was more influential in a shared meditative state as measured during hyperscanning. The absence of a directional connectivity metric in the present literature is a limitation to be discussed later in the paper.

## Study design breakdown

Seven of the studies met the inclusion criteria covering a wide range of experimental designs and study conditions: group-randomized guided mindfulness modalities, interoceptive breath-focused crossover experimental manipulations, expert joint meditation practice, open monitoring, and classroom-based group observations.

Two randomized controlled mindfulness studies examined guided mindfulness practices within different interactive tasks. One task in the study literature involved adult dyads who engaged in a cooperative interaction game (Deng et al., 2023). The task was computer-based, and adult dyads received shared feedback on success or failures during the game. Participants in the intervention group were asked to participate in a mindfulness exercise and listen to a 15-min audio-guided mindfulness recording before the interactive task, whereas participants in the control group engaged in quiet rest prior to the same task. In the other randomized experiment, adolescent dyads participated in an alternative task involving shared emotional picture viewing (Deng et al., 2023, 2024). Images were selected from emotionally salient datasets and designed to elicit arousal and emotional valence. Adolescent study participants were seated adjacent to each other, and one group was given a breath-centered guided mindfulness induction for moment-to-moment awareness, while the other sat quietly in rest before the emotional image viewing.

There were three crossover studies with an emphasis on interoceptive breathing and dyadic coordination with different task requests. In Balconi and Angioletti (2023), dyads coordinated using hand and finger movements in real time while they were instructed to pay attention to their breathing. This contrasted with a control group that had no coordination interaction but engaged in a verbal and cognitive matching task without breath emphasis. A variation of this design was employed in another crossover study that used goal setting during coordinated motor movements while comparing two conditions: a shared-goal condition (participants were told that task success depended on mutual coordination) and an individualized focus condition in which participants performed the same task with an individual focus. This was a within-subject crossover 2 × 2 design in which blocks were completed with and without interoceptive breath focus and with and without shared and unshared goals (Balconi and Angioletti, 2024). The third crossover design implemented a joint mindful breathing condition and compared conditions separating individuals and dyads (Coomans et al., 2021).

A hyperscanning observational study examined expert meditators using open monitoring compared to mind-wandering meditation in a co-presence condition (or meditators in the same room) as compared to a condition with meditators in different rooms (Matiz et al., 2021). The final study was a classroom study that compared breath-based meditation to a resting state and a music condition with the emphasis on larger network topology (Gao et al., 2025).

Across these study designs, interbrain synchrony outcomes were quantified with heterogenous methods and included differential outcomes for PLV, coherence within frequency domains, partial coherence, powered-based correlations, and metrics for comparative network topology. The following results are organized to categories based on frequency band to make meaningful comparisons between different experimental paradigms (see Table 3 for a complete summary).

**Table 3:** Summary of hyperscanning studies examining neural synchronization during mindfulness, interoceptive breath focus, and meditation.

Authors	Method	Sample	Brain region	Main results	Interpretation
Balconi and Angioletti (2023)	Interoceptive breath focus: short task-embedded prompts; adult dyads	Not a formal meditation block; explicit breath focus during task	Frontopolar, frontal midline, central; interbrain coherence (reported partial-correlation coherence) in α/θ/δ (continuous coordination)	Breath focus > No focus. Coherence, *p* = 0.01, increases in alpha (Fp1-Fp2, C3-C4) and delta/theta (Fz, C3-C4); effects larger in motor than linguistic synchrony.	Task-embedded interoceptive breath focus enhances frontal dyadic brain coherence, particularly during motor coordination. Motor timing may facilitate low-frequency neural synchrony beyond linguistic interaction.
Balconi and Angioletti (2024)	Interoceptive breath focus during a motor synchrony task +/– an explicit shared goal. 1-min blocks	13 dyads (adults); short, counterbalanced blocks: focus vs. no-focus × social (shared goal) vs. no-social; EEG + fNIRS hyperscanning	Left aPFC dominance; slow-band (δ/θ/α) EEG–fNIRS entrainment (interdyad) increases with breath focus and is boosted further by a shared goal	Breath focus + shared goal ↑ slow-band coupling, extending into left aPFC hemodynamics, δ: *p* = 0.01, coordination ↑ synchrony effect	Dyadic brain coupling in slow frequency bands is amplified when interoceptive focus is combined with an explicit shared goal, implicating left aPFC in intentional coordination and dyadic regulation.
Coomans et al. (2021)	Mindful breathing: joint vs. individual 3-min guided breathing blocks; adult dyads	Classic breat-focused attention; counterbalanced conditions	F8, T5/T6; interbrain coherence, α main effect (state), θ moderated by trait agreeableness (trait × condition).	Dyad > single, alpha coherence at F8/T5/T6 (*p* = 0.02 each); intersubject EEG coherence in frontotemporal (F8, T5, T6); alpha ↑ for joint > individual; theta at T6 ↑ with higher “agreeableness”	Dyadic mindful breathing increases alpha interbrain coherence relative to individual practice, and trait agreeableness moderates theta synchrony, indicating that both state and interpersonal traits influence dyadic neural alignment.
Deng et al. (2023)	Brief guided mindfulness: 15 min, audio guidance; adult dyads	Mindfulness vs. quiet rest control before task	Frontal > parietal–occipital; interbrain PLV, theta (event-locked feedback processing).	Mindfulness increased frontal theta PLV vs. Rest, *p* = 0.038, *post-hoc* success > failure *p* = 0.002, real dyad > surrogate dyad (*p* = 0.008)	Brief mindfulness induction enhances frontal theta phase synchrony during cooperative feedback processing, during successful joint outcomes, supporting a link between mindfulness and shared performance monitoring.
Deng et al. (2024)	Brief guided mindfulness: 20 min, audio guidance; adolescent dyads	Pre/post within-group + between-group (mindfulness vs. rest)	Frontal/central (valence-sensitive); interbrain PLV, gamma (shared emotional viewing).	Post-mindfulness ↑ in gamma PLV at frontal/central sites, post-mindfulness increases: Fz vs. pre *p* = 0.007	Mindfulness modulates gamma-band interbrain synchrony during shared emotional processing, suggesting enhanced socio-affective alignment independent of self-reported arousal changes.
Gao et al. (2025)	Short classroom meditation 5 min per condition; classroom group vs. music	Group setting; simple breath meditation block before/alongside tasks	Single-channel Fp1 per student; group network topology (clustering, small-world), not dyadic PLV/coherence.	Music produced highest pairwise α correlations (common-input) meditation ↑ clustering and small-worldness vs. rest (network integration).	In classroom settings, breath meditation alters group-level network topology rather than simple pairwise coherence, indicating that contemplative practice may influence large-scale integration beyond dyadic coupling.
Matiz et al. (2021)	Open-monitoring meditation (MOM) vs. mind wandering; 7-min blocks, expert couples	Expert-level practice; no explicit breath cue, OM-style mindfulness	Fronto-limbic Independent Component (IC) clusters (mesial PFC/ACC/insula-connected); co-activation (IC power) slow-band co-activation in experts.	During open-monitoring meditation vs. mind wandering, right frontolimbic cluster ↑ <0.01. Dyad > individual, *p* < 0.005. Frontolimbic gamma and alpha, theta, delta. IC-cluster power ↑	Expert meditators exhibit increased frontolimbic gamma and slow-band co-activation during co-presence, suggesting shared contemplative expertise strengthens neural coordination beyond individual meditation alone.

## Findings across spectral windows

### Theta band interbrain synchrony

Theta interbrain synchrony was one of the most reproducible signatures, appearing in five of seven studies. In the adult dyad study of brief mindfulness, there was an increase in frontal theta PLV during feedback processing that took place during the cooperative game when compared to the rest condition. The finding was frontal dominant and exhibited a stronger PLV for success vs. failure trials in the breath-based body scan group. Theta synchrony was strongest during shared success trials (both participants coordinated successfully during different computer tasks) compared to the shared failure (dyads failed to coordinate successfully). Also, the outcome was strongest in the mindfulness sample (Deng et al., 2023). The data suggest mindfulness amplified the effect of interpersonal connectivity and joint cognitive processing. Also, real dyads elicited greater synchrony than surrogate (random) dyads, one of the strongest controls since it suggested true coupling had taken place beyond the influence of the shared task (Deng et al., 2023).

In the breath-focused dyadic motor synchrony, results indicated theta coherence had increased over frontal-central neural regions. This finding was salient relative to other study tasks such as the noninteroceptive mindful breathing group and a linguistic task condition control group in which participants completed a verbal task related to placing words semantically together within a similar category (Balconi and Angioletti, 2023).

During joint mindful breathing, theta coherence localized to right temporal sites increased in more “agreeable” dyads. This finding highlights that certain interpersonal traits can modulate the power of topography within interbrain clusters (Coomans et al., 2021). For expert meditators practicing together, there was also a low-frequency theta cluster effect when partners practiced together compared to when practiced individually (Matiz et al., 2021). The outcome suggested a context-contingent finding related to meditator expertise for theta spectral windows. The theta findings suggest frontal and central theta as a salient neural marker of shared monitoring of breath, adaptive executive control, and sensory-linked motor coordination within contemplative paradigms in the review (Kelso, 1995).

### Alpha band interbrain synchrony

The alpha interbrain synchrony findings were increasingly evident in motor coordination tasks that used interoceptive breath focus. Alpha findings were localized in frontal and central brain regions. For the interoceptive breath focus and dyadic motor coordination tasks synchrony increased at frontopolar and central electrodes compared to the linguistic control (Balconi and Angioletti, 2023). In another study examining joint mindful breathing practice as measured in dyads, there was increased alpha coherence in frontotemporal sites when contrasted with individual participants breathing alone (Coomans et al., 2021). Another alpha finding was with an experienced meditator cohort. When expert meditators practiced together in the same room, they elicited stronger activation than when they were practicing separately. The author suggested that that context and individual expertise potentiated alpha band synchrony metrics (Matiz et al., 2021). The alpha findings across studies indicate variable outcomes across bands and regions. When motor tasks and breath focus were combined the alpha neural synchrony findings were increasingly salient.

### Gamma band interbrain synchrony

Gamma oscillations have long been proposed as a mechanism for large-scale cognitive integration and neuronal communication through coherence; however, gamma interbrain synchrony was not as common, appearing in only two of the seven studies, and was specific to context (Fries, 2005; Singer, 1999). In an adolescent population, gamma effects appeared in frontal-central regions and appeared as higher gamma PLVs within dyads during shared emotional picture viewing after a mindfulness-based induction (Deng et al., 2024). The effect was not due to changes in arousal as arousal self-report ratings decreased after the mindfulness intervention.

In experienced meditator dyads, open monitoring meditation is associated with increased gamma band co-activation localized to frontal and frontolimbic regions. The gamma power increases were significantly higher when partners practiced together compared to them practicing in separate rooms suggesting a context-specific modulation. This increase was indicated in the open monitoring condition and not observed during the mind wandering baseline. Also, gamma effects reflect coordinated power increases rather than phase locking. The results suggest that advanced training potentiates neural binding with larger groups, beyond individual practice alone (Matiz et al., 2021).

### Delta interbrain synchrony

Delta effects were the least examined but emerged in a specific study with interoceptive breath focus and shared goals. The delta synchrony effects were not observed in several study paradigms and were absent in induction and shared affect tasks. Within EEG–FNIRS hyperscanning during breath-focused motor coordination delta band entrainment increased in dyads with shared goal conditions and regionally localized to left anterior PFC (aPFC) (Balconi and Angioletti, 2024; Yücel et al., 2021).

Akin to earlier findings for higher-frequency spectral bands, expert meditators exhibited an increase in delta band co-activation while practicing together (Matiz et al., 2021). The low-frequency result suggests potential coupling of autonomic systems and cortical dynamics during contemplative states (Balconi and Angioletti, 2023; Balconi et al., 2023). Delta activation is often associated with metabolic and autonomic processes, and these may mediate components of interpersonal connection. This speculative link could be explored within future contemplative hyperscanning studies (Palumbo et al., 2017; Park and Thayer, 2014).

## Limitations

Despite this review's synthesis, several limitations prevent drawing conclusions based on it. The literature base is underpowered, and there are substantial differences across all studies. Heterogeneity in study designs and variability in protocol implementations are limitations that impact direct comparisons between findings and limit generalizability. Furthermore, there are limits to the operational definition of mindfulness such that nominally similar practices are different in their actual implementation, and this variability can affect reproducibility and study validity. For example, open monitoring, body scanning, and breath awareness are not identical and involve different breath-based timing and attentional anchors.

Another issue is the reporting of synchronization protocols in several studies and the absence of surrogate and time-shift nulls. Reporting issues include disclosure of jitter values, time latency, and clock drift between systems. Comparisons with time-shift nulls and adequate reporting would provide important controls for spurious coupling outcomes and help delineate between artifacts and a true dyadic alignment (Burgess, 2013; Theiler et al., 1992). An appraisal of study quality for the manuscript determined that most studies were rated low to fair risk of bias. Small sample sizes and an inconsistent null-model implementation represented the most pronounced methodological limitations.

Comparisons between different connectivity metrics may also obfuscate meaningful contrasts (Bastos and Schoffelen, 2016). Connectivity metrics provide data on synchrony; however, they do not capture interbrain directionality or asymmetry. It is not clear whether dyadic interbrain interactions occur spontaneously or are propagated by a leading cognitive source that is more dominant in the interplay between neural sources. Granger causality metrics provide estimates of temporal interplay that were not examined in many studies (Seth et al., 2015; Friston et al., 2014).

## Conclusion

This narrative review synthesizes the emerging literature examining hyperscanning during mindfulness and meditation. Across eligible studies spanning different populations and protocols, synchrony was observed primarily in the delta, theta, alpha, and gamma bands, with the most consistent localization in frontal, frontocentral, and frontotemporal regions. Condition-dependent effects emerged in the gamma and delta bands; however, findings varied across spectral windows.

Task-based breath focus on a motor activity globally enhanced synchrony between people within the alpha, theta, and delta bands (Balconi and Angioletti, 2023). Mindfulness increased gamma synchrony between participants during emotional viewing tasks in frontal regions (Deng et al., 2024). In contrast to higher-frequency effects, dyadic coupling in lower bands was potentiated when breath focus was combined with a shared goal (Balconi and Angioletti, 2024). In related interactive tasks, mindfulness increased theta phase synchrony during cooperative games with shared feedback. Positive performance feedback further enhanced synchrony (Deng et al., 2023). Similarly, mindful breathing increased coherence between individuals in frontal and temporal brain regions and was moderated by personality traits.

For example, agreeableness was found to be a moderating trait variable and higher agreeableness was associated with increased dyadic alpha coherence during the joint mindful breathing practice localized to frontotemporal regions (Coomans et al., 2021). These findings suggest that trait characteristics may influence interpersonal connectivity and warrant further investigation, particularly within mindfulness-related practices. One relevant domain is awareness and attitudinal modulation, core components of contemporary mindfulness and Mindfulness Based Stress Reduction (MBSR) programs (Kabat-Zinn, 1990, 2003; Lutz et al., 2008). Agreeableness combined with positive shared feedback may further potentiate synchronization between meditators; however, this interaction has not yet been directly tested.

Another finding was increased individual EEG spectral power during group meditation compared to solitary practice, particularly in shared or dyadic conditions. The potential effects of collective meditation have long been discussed in contemplative literature, including claims regarding broader social impact (Hagelin et al., 1999). However, empirical neurophysiological data examining the biological basis of group meditation effects remain limited. One possible interpretation is that as more individuals engage in temporally aligned neural activity, aggregate coherence may increase at the group level. While speculative, this raises the question of whether shared contemplative states potentiate neural synchrony more strongly in groups than during solitary practice.

Hyperscanning has rapidly evolved over the last decade. With the advent of open-source EEG hardware in 2013, studies have shifted from proof-of-concept work to increasingly complex ecological tasks across disciplines (Czeszumski et al., 2020; Montague et al., 2002). Recent methodological advances extend beyond PLV and coherence to include multibrain “microstates,” which may capture aspects of meditation phenomenology such as stabilization, mind wandering, deeper focus, and real-time connectivity dynamics (Tognoli and Kelso, 2014; Dumas et al., 2010). This progression is further reflected in the increased application of time-domain Granger causality and other temporally resolved connectivity metrics (Granger, 1969; Seth et al., 2015).

The interplay of temporal dynamics represents a shift away from traditional shared-input paradigms that characterize much of the hyperscanning literature, in which participants receive a common stimulus and neural outcomes are examined primarily as a function of shared input. A shared regulation paradigm would instead require more complex temporal analyses to examine reciprocal and dyadic feedback processes between interacting individuals and potentially even more complex group dynamics (Hasson et al., 2012; Schilbach et al., 2013). Although such paradigms are methodologically more demanding, recent advances in analytic tools and the increasing availability of more powerful computational resources may make these approaches increasingly feasible in the coming years (Gramfort et al., 2014; Pernet et al., 2019).

Importantly, examining temporal interplay may also allow closer integration of neural synchrony with self-report and psychometric data. For example, in adolescent dyads, mindfulness was associated with greater interbrain synchrony compared to control conditions, and relational traits such as agreeableness moderated the magnitude of dyadic synchrony during joint mindful breathing, particularly within theta-band temporal dynamics (Coomans et al., 2021). Although this study employed a shared-input design, similar protocols could be adapted into shared-regulation paradigms to examine not only temporal neural dynamics between individuals, but also how self-report and physiological state-based measures fluctuate and interact over the course of a session.

The notion of a “temporal interplay” framework may relate to existing cognitive theories and a predictive processing heuristic within social coordination (Friston, 2010; Hasson et al., 2012; Friston et al., 2014). For example, “highly agreeable” partners may call upon brain networks that can anticipate each other's timing, intentions, and even actions and as a result attenuate latency of coordination during dyadic breathing. In predictive-processing theory, Friston (2010) states that the neural system manifests and updates cognitive heuristics to decrease prediction errors and build a predictive feedback loop between self and other. This contemporary notion of a cognitive interplay between systems can be traced back to ancient spiritual texts related to Buddhism and mindfulness. The *pratityasamutpāda* from the fourth century states that all phenomena arise in unique and conditional interdependence to each other, and the concept is perhaps a meaningful historical analog to contemporary temporal neural interdependence (Dogen, 1233/1975; Thich Nhat Hanh, 1998a,b). As mindfulness-based neural hyperscanning continues to expand, researchers may further develop this fascinating frontier science to reveal the nuanced patterns and complexities within human connectivity research and the true nature of contemplative minds.

## References

[B1] Astolfi L. Cincotti F. Mattia D. De Vico Fallani F. Salinari S. Vecchiato G. . (2011). Imaging the social brain by simultaneous hyperscanning during subject interaction. IEEE Intell. Syst. 25, 38–45. doi: 10.1109/MIS.2011.61 22287939 PMC3267574

[B2] Astolfi L. Toppi J. De Vico Fallani F. Vecchiato G. Cincotti F. Wilke C. . (2010). Neuroelectrical hyperscanning measures simultaneous brain activity in humans. Brain Topogr. 23, 243–256. doi: 10.1007/s10548-010-0147-9 20480221

[B3] Babiloni F. Astolfi L. Cincotti F. Mattia D. Tocci A. Tarantino A. . (2012). Cortical activity and connectivity of human brain during the prisoner's dilemma: an EEG hyperscanning study. Front. Hum. Neurosci. 6:131. doi: 10.3389/fnhum.2012.00131 18003118

[B4] Balconi M. Allegretta R. A. Angioletti L. (2023). Autonomic synchrony induced by hyperscanning interoception during interpersonal synchronization tasks. Front. Hum. Neurosci. 17:1200750. doi: 10.3389/fnhum.2023.1200750 37545591 PMC10400890

[B5] Balconi M. Angioletti L. (2023). Dyadic inter-brain EEG coherence induced by interoceptive hyperscanning. Sci. Rep. 13:4344. doi: 10.1038/s41598-023-31494-y 36927763 PMC10020471

[B6] Balconi M. Angioletti L. (2024). Inter-brain entrainment (IBE) during interoception: a multimodal EEG-fNIRS coherence-based hyperscanning approach. Neurosci. Lett. 831:137789. doi: 10.1016/j.neulet.2024.137789 38670524

[B7] Balconi M. Vanutelli M. E. (2017). Cooperation and competition with hyperscanning methods: review and future application to emotion domain. Front. Comput. Neurosci. 11:86. doi: 10.3389/fncom.2017.00086 29033810 PMC5627061

[B8] Bastos A. M. Schoffelen J.-M. (2016). A tutorial review of functional connectivity analysis methods and their interpretational pitfalls. Front. Syst. Neurosci. 9:175. doi: 10.3389/fnsys.2015.00175 26778976 PMC4705224

[B9] Bevilacqua D. Davidesco I. Wan L. Chaloner K. Rowland J. Ding M. . (2019). Brain-to-brain synchrony and learning outcomes vary by student–teacher dynamics: evidence from a real-world classroom EEG study. J. Cogn. Neurosci. 31, 401–411. doi: 10.1162/jocn_a_01274 29708820

[B10] Brewer J. A. Worhunsky P. D. Gray J. R. Tang Y.-Y. Weber J. Kober H. . (2011). Meditation experience is associated with differences in default mode network activity and connectivity. Proc. Natl. Acad. Sci. U.S.A. 108, 20254–20259. doi: 10.1073/pnas.1112029108 22114193 PMC3250176

[B11] Burgess A. P. (2013). On the interpretation of synchronization in EEG hyperscanning studies: a cautionary note. Front. Hum. Neurosci. 7:881. doi: 10.3389/fnhum.2013.00881 24399948 PMC3870947

[B12] Cahn B. R. Polich J. (2006). Meditation states and traits: EEG, ERP, and neuroimaging studies. Psychol. Bull. 132, 180–211. doi: 10.1037/0033-2909.132.2.180 16536641

[B13] Carlson L. E. Speca M. Patel K. D. Goodey E. (2007). Mindfulness-based stress reduction in relation to quality of life, mood, symptoms of stress, and immune parameters in breast and prostate cancer outpatients. Psychosom. Med. 65, 571–581. doi: 10.1097/01.PSY.0000074003.35911.41 12883107

[B14] Carson J. W. Carson K. M. Gil K. M. Baucom D. H. (2004). Mindfulness-based relationship enhancement. Behav. Ther. 35, 471–494. doi: 10.1016/S0005-7894(04)80028-5

[B15] Cohen M. X. (2014). Analyzing Neural Time Series Data: Theory and Practice. Cambridge, MA: MIT Press. doi: 10.7551/mitpress/9609.001.0001

[B16] Coomans E. Geraedts I. K. Deijen J. B. Keeser D. Pogarell O. Engelbregt H. J. (2021). Intersubject EEG coherence in healthy dyads during individual and joint mindful breathing exercise: an EEG-based experimental hyperscanning study. Adv. Cogn. Psychol. 17, 250–260. doi: 10.5709/acp-0334-7

[B17] Cui X. Bryant D. M. Reiss A. L. (2012). NIRS-based hyperscanning reveals increased interpersonal coherence in superior frontal cortex during cooperation. Neuroimage 59, 2430–2437. doi: 10.1016/j.neuroimage.2011.09.003 21933717 PMC3254802

[B18] Czeszumski A. Eustergerling S. Lang A. Menrath D. Gerstenberger M. Schuberth S. . (2020). Hyperscanning: a valid method to study inter-brain underpinnings of social interaction. Front. Hum. Neurosci. 14:39. doi: 10.3389/fnhum.2020.00039 32180710 PMC7059252

[B19] Dai X. Li X. Xia N. Xi J. Zhang Y. (2023). Client-counselor behavioral and inter-brain synchronization among dismissing and secure clients and its association with alliance quality and outcome. Psychother. Res. 34, 1103–1116. doi: 10.1080/10503307.2023.2249587 37643580

[B20] Delorme A. Makeig S. (2004). EEGLAB: an open source toolbox for analysis of single-trial EEG dynamics including independent component analysis. J. Neurosci. Methods 134, 9–21. doi: 10.1016/j.jneumeth.2003.10.009 15102499

[B21] Deng X. Lin M. Li X. (2024). Mindfulness meditation enhances interbrain synchrony of adolescents when experiencing different emotions simultaneously. Cereb. Cortex 34:bhad474. doi: 10.1093/cercor/bhad474 38061691

[B22] Deng X. Yang M. Chen X. Zhan Y. (2023). The role of mindfulness on theta inter-brain synchrony during cooperation feedback processing: an EEG-based hyperscanning study. Int. J. Clin. Health Psychol. 23:100396. doi: 10.1016/j.ijchp.2023.100396 37521502 PMC10372402

[B23] Dikker S. Wan L. Davidesco I. Kaggen L. Oostrik M. McClintock J. . (2017). Brain-to-brain synchrony tracks real-world group interactions in the classroom. Curr. Biol. 27, 1375–1380. doi: 10.1016/j.cub.2017.04.002 28457867

[B24] Dogen (1233/1975). Shobogenzo [*Treasury of the True Dharma Eye*]. Transl. by K. Tanahashi. Berkeley, CA: Shambhala.

[B25] Dumas G. Nadel J. Soussignan R. Martinerie J. Garnero L. (2010). Inter-brain synchronization during social interaction. PLoS ONE 5:e12166. doi: 10.1371/journal.pone.0012166 20808907 PMC2923151

[B26] Fox K. C. R. Dixon M. L. Nijeboer S. Girn M. Floman J. L. Lifshitz M. . (2016). Functional neuroanatomy of meditation: a review and meta-analysis of 78 fMRI studies. Neurosci. Biobehav. Rev. 65, 208–228. doi: 10.1016/j.neubiorev.2016.03.021 27032724

[B27] Fredrickson B. L. Cohn M. A. Coffey K. A. Pek J. Finkel S. M. (2008). Open hearts build lives: positive emotions, induced through loving-kindness meditation, build consequential personal resources. J. Pers. Soc. Psychol. 95, 1045–1062. doi: 10.1037/a0013262 18954193 PMC3156028

[B28] Fries P. (2005). A mechanism for cognitive dynamics: neuronal communication through neuronal coherence. Trends Cogn. Sci. 9, 474–480. doi: 10.1016/j.tics.2005.08.011 16150631

[B29] Friston K. (2010). The free-energy principle: a unified brain theory? Nat. Rev. Neurosci. 11, 127–138. doi: 10.1038/nrn2787 20068583

[B30] Friston K. J. Bastos A. M. Litvak V. Stephan K. E. Fries P. Moran R. J. . (2014). DCM for complex-valued data: cross-spectra, coherence and phase-delays. Neuroimage 59, 439–455. doi: 10.1016/j.neuroimage.2011.07.048 21820062 PMC3200431

[B31] Gao J. Leung H. K. Lee K. C. Poon C. C. Huang G. Liao J. . (2025). Inter-brain synchronization in classroom during high-entropy music listening and meditation: a hyperscanning EEG study. Front. Neurosci. 19:1557904. doi: 10.3389/fnins.2025.1557904 40313539 PMC12044879

[B32] Geweke J. (1982). Measurement of linear dependence and feedback between multiple time series. J. Am. Stat. Assoc. 77, 304–313. doi: 10.1080/01621459.1982.10477803

[B33] Goyal M. Singh S. Sibinga E. M. S. Gould N. F. Rowland-Seymour A. Sharma R. . (2014). Meditation programs for psychological stress and well-being: a systematic review and meta-analysis. JAMA Intern. Med. 174, 357–368. doi: 10.1001/jamainternmed.2013.13018 24395196 PMC4142584

[B34] Gramfort A. Luessi M. Larson E. Engemann D. A. Strohmeier D. Brodbeck C. . (2014). MNE software for processing MEG and EEG data. Neuroimage 86, 446–460. doi: 10.1016/j.neuroimage.2013.10.027 24161808 PMC3930851

[B35] Granger C. W. J. (1969). Investigating causal relations by econometric models and cross-spectral methods. Econometrica 37, 424–438. doi: 10.2307/1912791

[B36] Hagelin J. S. Rainforth M. V. Cavanaugh K. L. Alexander C. N. Shatkin S. F. Orme-Johnson D. W. . (1999). Effects of group practice of the Transcendental Meditation program on preventing violent crime in Washington, D.C.: results of the National Demonstration Project, June–July 1993. Soc. Indic. Res. 47, 153–201. doi: 10.1023/A:1006978911496

[B37] Hasson U. Ghazanfar A. A. Galantucci B. Garrod S. Keysers C. (2012). Brain-to-brain coupling: a mechanism for creating and sharing a social world. Trends Cogn. Sci. 16, 114–121. doi: 10.1016/j.tics.2011.12.007 22221820 PMC3269540

[B38] Hutcherson C. A. Seppala E. M. Gross J. J. (2008). Loving-kindness meditation increases social connectedness. Emotion 8, 720–724. doi: 10.1037/a0013237 18837623

[B39] Jha A. P. Krompinger J. Baime M. J. (2007). Mindfulness training modifies subsystems of attention. Cogn. Affect. Behav. Neurosci. 7, 109–119. doi: 10.3758/CABN.7.2.109 17672382

[B40] Kabat-Zinn J. (1990). Full Catastrophe Living: Using the Wisdom of Your Body and Mind to Face Stress, Pain, and Illness. New York, NY: Delacorte Press.

[B41] Kabat-Zinn J. (2003). Mindfulness-based interventions in context: past, present, and future. Clin. Psychol. Sci. Pract. 10, 144–156. doi: 10.1093/clipsy.bpg016

[B42] Kelso J. A. S. (1995). Dynamic Patterns: The Self-Organization of Brain and Behavior. Cambridge, MA: MIT Press.

[B43] King-Casas B. Tomlin D. Anen C. Camerer C. F. Quartz S. R. Montague P. R. (2005). Getting to know you: reputation and trust in a two-person economic exchange. Science 308, 78–83. doi: 10.1126/science.1108062 15802598

[B44] Koike T. Tanabe H. C. Sadato N. (2016). Hyperscanning neuroimaging technique to reveal the “two-in-one” system in social interactions. Neurosci. Res. 90, 25–32. doi: 10.1016/j.neures.2014.11.006 25499683

[B45] Kok B. E. Singer T. (2017). Effects of contemplative dyads on engagement and perceived social connectedness over 9 months of mental training: A randomized clinical trial. JAMA Psych. 74, 126–134. doi: 10.1001/jamapsychiatry.2016.3360 28030741

[B46] Lachaux J.-P. Rodriguez E. Martinerie J. Varela F. J. (1999). Measuring phase synchrony in brain signals. Hum. Brain Mapp. 8, 194–208. doi: 10.1002/(sici)1097-0193(1999)8:4<194::aid-hbm4>3.0.co;2-c 10619414 PMC6873296

[B47] Lindenberger U. Li S.-C. Gruber W. Müller V. (2009). Brains swinging in concert: Cortical phase synchronization while playing guitar. BMC Neurosci. 10:22. doi: 10.1186/1471-2202-10-22 19292892 PMC2662862

[B48] Lindsay E. K. Young S. Smyth J. M. Brown K. W. Creswell J. D. (2019). Mindfulness training reduces loneliness and increases social contact in a randomized controlled trial. Proc. Natl. Acad. Sci. U.S.A. 116, 3488–3493. doi: 10.1073/pnas.1813588116 30808743 PMC6397548

[B49] Liu Y. Piazza E. A. Simony E. . (2018). Measuring speaker–listener neural coupling with functional near-infrared spectroscopy. Sci. Rep. 8:420. doi: 10.1038/srep43293 28240295 PMC5327440

[B50] Lutz A. Greischar L. L. Rawlings N. B. Ricard M. Davidson R. J. (2004). Long-term meditators self-induce high-amplitude gamma synchrony during mental practice. Proc. Natl. Acad. Sci. U.S.A. 101, 16369–16373. doi: 10.1073/pnas.0407401101 15534199 PMC526201

[B51] Lutz A. Slagter H. A. Dunne J. D. Davidson R. J. (2008). Attention regulation and monitoring in meditation. Trends Cogn. Sci. 12, 163–169. doi: 10.1016/j.tics.2008.01.005 18329323 PMC2693206

[B52] Maris E. Oostenveld R. (2007). Nonparametric statistical testing of EEG- and MEG-data. J. Neurosci. Methods 164, 177–190. doi: 10.1016/j.jneumeth.2007.03.024 17517438

[B53] Matiz A. Crescentini C. Bergamasco M. Budai R. Fabbro F. (2021). Inter-brain co-activations during mindfulness meditation. Implications for devotional and clinical settings. Conscious. Cogn. 95:103210. doi: 10.1016/j.concog.2021.103210 34562699

[B54] Montague P. R. Berns G. S. Cohen J. D. McClure S. M. Pagnoni G. Dhamala M. . (2002). Hyperscanning: simultaneous fMRI during linked social interactions. Neuroimage 16, 1159–1164. doi: 10.1006/nimg.2002.1150 12202103

[B55] National Heart Lung, and Blood Institute. (2014). Study Quality Assessment Tools. U.S. Department of Health and Human Services. Available online at: https://www.nhlbi.nih.gov/health-topics/study-quality-assessment-tools (Accessed October 1, 2025).

[B56] Noah J. A. Zhang X. Dravida S. Ono Y. Naples A. McPartland J. C. . (2020). Real-time eye-to-eye contact is associated with cross-brain neural coupling in angular gyrus. Front. Hum. Neurosci. 14:19. doi: 10.3389/fnhum.2020.00019 32116606 PMC7016046

[B57] Nolte G. Bai O. Wheaton L. Mari Z. Vorbach S. Hallett M. . (2004). Identifying true brain interaction from EEG data using the imaginary part of coherency. Clin. Neurophysiol. 115, 2292–2309. doi: 10.1016/j.clinph.2004.04.029 15351371

[B58] Page M. J. McKenzie J. E. Bossuyt P. M. Boutron I. Hoffmann T. C. Mulrow C. D. . (2021). The PRISMA 2020 statement: an updated guideline for reporting systematic reviews. BMJ 372:n71. doi: 10.1136/bmj.n71 33782057 PMC8005924

[B59] Palumbo R. V. Marraccini M. E. Weyandt L. L. Wilder-Smith O. McGee H. A. Liu S. . (2017). Interpersonal autonomic physiology: a systematic review of the literature. Pers. Soc. Psychol. Rev. 21, 99–141. doi: 10.1177/1088868316628405 26921410

[B60] Park G. Thayer J. F. (2014). From the heart to the mind: cardiac vagal tone modulates top-down and bottom-up visual perception and attention to emotional stimuli. Front. Psychol. 5:278. doi: 10.3389/fpsyg.2014.00278 24817853 PMC4013470

[B61] Pernet C. R. Garrido M. I. Gramfort A. Maurits N. Michel C. M. Oostenveld R. . (2019). EEG-BIDS, an extension to the brain imaging data structure for EEG. Sci. Data 6:103. doi: 10.1038/s41597-019-0104-8 31239435 PMC6592877

[B62] Sänger J. Müller V. Lindenberger U. (2012). Intra- and inter-brainsynchronization and network properties when playing guitar in duets. Front. Hum. Neurosci. 6:312. doi: 10.3389/fnhum.2012.00312 23226120 PMC3509332

[B63] Schilbach L. Timmermans B. Reddy V. Costall A. Bente G. Schlicht T. . (2013). Toward a second-person neuroscience. Behav. Brain Sci. 36, 393–414. doi: 10.1017/S0140525X12000660 23883742

[B64] Segal Z. V. Williams J. M. G. Teasdale J. D. (2010). Mindfulness-based cognitive therapy for depression (2nd ed.). New York, NY: Guilford Press.

[B65] Seth A. K. Barrett A. B. Barnett L. (2015). Granger causality analysis in neuroscience and neuroimaging. J. Neurosci. 35, 3293–3297. doi: 10.1523/JNEUROSCI.4399-14.2015 25716830 PMC4339347

[B66] Singer W. (1999). Neuronal synchrony: a versatile code for the definition of relations? Neuron 24, 49–65, 111–125. doi: 10.1016/S0896-6273(00)80821-1 10677026

[B67] Spinoza B. (1677/1996). Ethics. Transl. by E. Curley. London: Penguin Books. (Original work published 1677).

[B68] Sterne J. A. C. Savović J. Page M. J. Elbers R. G. Blencowe N. S. Boutron I. . (2019). RoB 2: a revised tool for assessing risk of bias in randomized trials. BMJ 366:l4898. doi: 10.1136/bmj.l4898 31462531

[B69] Tang Y.-Y. Hölzel B. K. Posner M. I. (2015). The neuroscience of mindfulness meditation. Nat. Rev. Neurosci. 16, 213–225. doi: 10.1038/nrn3916 25783612

[B70] Tang Y.-Y. Ma Y. Wang J. Fan Y. Feng S. Lu Q. . (2007). “Short-term meditation training improves attention and self-regulation” in Proceedings of the National Academy of Sciences 104, 17152–17156. doi: 10.1073/pnas.0707678104 17940025 PMC2040428

[B71] TenHouten W. Schussel L. Gritsch M. F. Kaplan C. D. (2023). Hyperscanning and the future of neurosociology. Sociol. Methodol. 53, 139–157. doi: 10.1177/00811750221128790

[B72] Theiler J. Eubank S. Longtin A. Galdrikian B. Farmer J. D. (1992). Testing for nonlinearity in time series: the method of surrogate data. Physica D 58, 77–94. doi: 10.1016/0167-2789(92)90102-S

[B73] Thich Nhat Hanh (1998a). Interbeing: Fourteen Guidelines for Engaged Buddhism. Berkeley, CA: Parallax Press.

[B74] Thich Nhat Hanh (1998b). The Heart of the Buddha's Teaching: Transforming Suffering into Peace, Joy, and Liberation. Portland, OR: Broadway Books.

[B75] Tognoli E. Kelso J. A. S. (2014). The metastable brain. Neuron 81, 35–48. doi: 10.1016/j.neuron.2013.12.022 24411730 PMC3997258

[B76] Yücel M. A. Lühmann A. V. Scholkmann F. Gervain J. Dan I. Ayaz H. . (2021). Best practices for fNIRS publications (COBRA): recommendations. Neurophotonics 8:012101. doi: 10.1117/1.NPh.8.1.019802 33442557 PMC7793571

[B77] Zhang Y. Meng T. Yang Y. Hu Y. (2020). Experience-dependent counselor-client brain synchronization during psychological counseling. eNeuro 7:ENEURO.0236-20.2020. doi: 10.1523/ENEURO.0236-20.2020 32878962 PMC7519169

